# Paired inspiratory-expiratory chest CT scans to assess for small airways disease in COPD

**DOI:** 10.1186/1465-9921-14-42

**Published:** 2013-04-08

**Authors:** Craig P Hersh, George R Washko, Raúl San José Estépar, Sharon Lutz, Paul J Friedman, MeiLan K Han, John E Hokanson, Philip F Judy, David A Lynch, Barry J Make, Nathaniel Marchetti, John D Newell, Frank C Sciurba, James D Crapo, Edwin K Silverman

**Affiliations:** 1 Channing Division of Network Medicine, Brigham and Women’s Hospital and Harvard Medical School, 181 Longwood Avenue, Boston, MA, 02115, USA; 2 Division of Pulmonary and Critical Care Medicine, Brigham and Women’s Hospital and Harvard Medical School, Boston, MA, USA; 3 Department of Biostatistics, Colorado School of Public Health, Aurora, CO, USA; 4 Department of Epidemiology, Colorado School of Public Health, Aurora, CO, USA; 5 Department of Radiology, University of California, San Diego, CA, USA; 6 Division of Pulmonary and Critical Care Medicine, University of Michigan Health System, Ann Arbor, MI, USA; 7 Department of Radiology, Brigham and Women’s Hospital and Harvard Medical School, Boston, MA, USA; 8 Department of Radiology, National Jewish Health, Denver, CO, USA; 9 Division of Pulmonary and Critical Care Medicine, National Jewish Health, Denver, CO, USA; 10 Section of Pulmonary and Critical Care Medicine, Temple University, Philadelphia, PA, USA; 11 Department of Radiology, University of Iowa, Iowa City, IA, USA; 12 Division of Pulmonary, Allergy, and Critical Care Medicine, University of Pittsburgh, Pittsburgh, PA, USA

**Keywords:** Emphysema, Chest CT scan, Small airways, Lung function tests, Smoking

## Abstract

**Background:**

Gas trapping quantified on chest CT scans has been proposed as a surrogate for small airway disease in COPD. We sought to determine if measurements using paired inspiratory and expiratory CT scans may be better able to separate gas trapping due to emphysema from gas trapping due to small airway disease.

**Methods:**

Smokers with and without COPD from the COPDGene Study underwent inspiratory and expiratory chest CT scans. Emphysema was quantified by the percent of lung with attenuation < −950HU on inspiratory CT. Four gas trapping measures were defined: (1) Exp_−856_, the percent of lung < −856HU on expiratory imaging; (2) E/I MLA, the ratio of expiratory to inspiratory mean lung attenuation; (3) RVC_856-950_, the difference between expiratory and inspiratory lung volumes with attenuation between −856 and −950 HU; and (4) Residuals from the regression of Exp_−856_ on percent emphysema.

**Results:**

In 8517 subjects with complete data, Exp_−856_ was highly correlated with emphysema. The measures based on paired inspiratory and expiratory CT scans were less strongly correlated with emphysema. Exp_−856_, E/I MLA and RVC_856-950_ were predictive of spirometry, exercise capacity and quality of life in all subjects and in subjects without emphysema. In subjects with severe emphysema, E/I MLA and RVC_856-950_ showed the highest correlations with clinical variables.

**Conclusions:**

Quantitative measures based on paired inspiratory and expiratory chest CT scans can be used as markers of small airway disease in smokers with and without COPD, but this will require that future studies acquire both inspiratory and expiratory CT scans.

## Introduction

**Figure 1:**
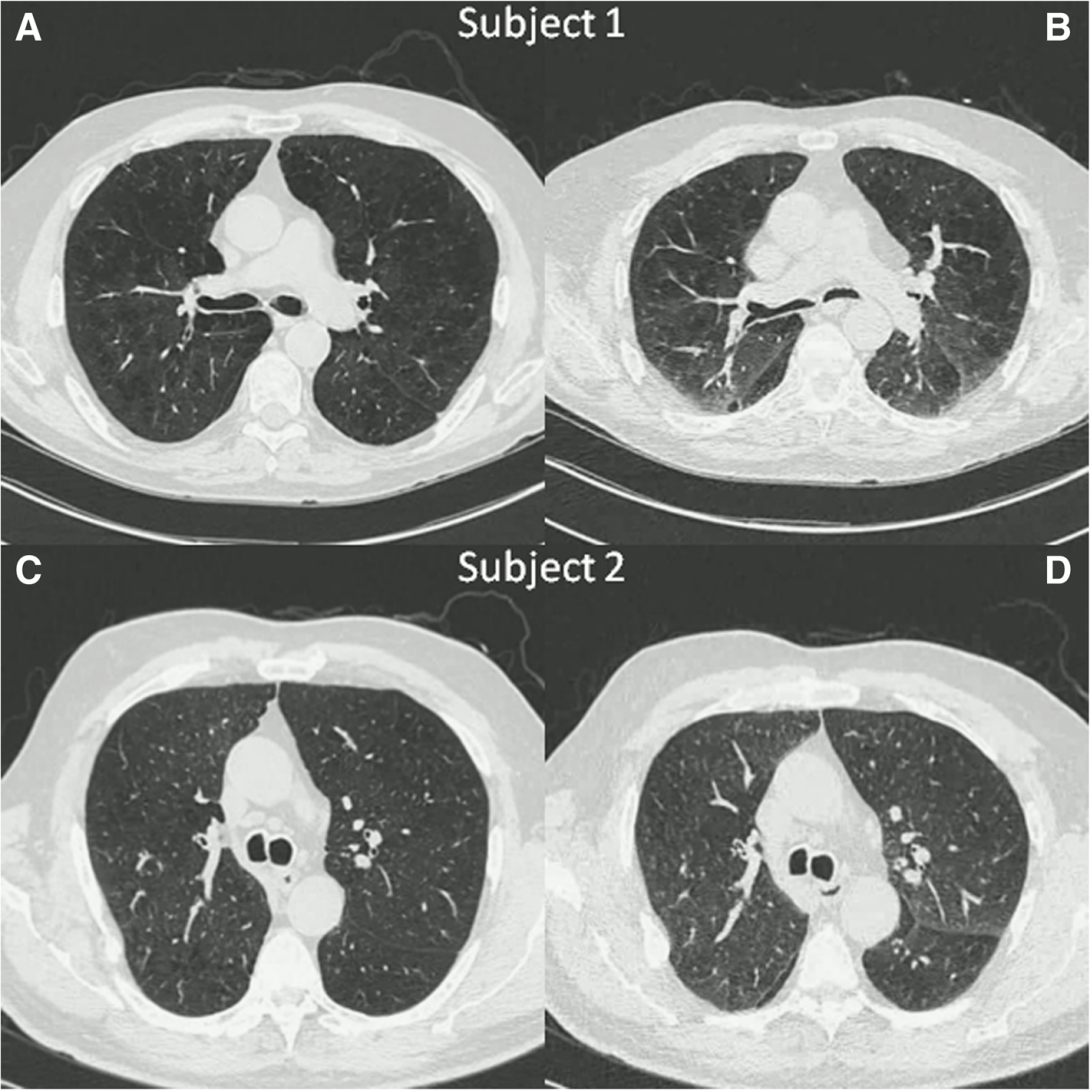
**Inspiratory (A,C) and expiratory (B,D) chest CT images from two subjects with similar percent of lung voxels with attenuation values < −856HU on expiratory CT scans (Exp**_**−856**_**) demonstrate the inability of expiratory image analysis alone to distinguish gas trapping due to emphysema from gas trapping due to small airway disease.** Subject 1: Exp_−856_ = 38.5%, percent emphysema (Insp_−950_) = 23.1%. Subject 2: Exp_−856_ = 37.1%, Insp_−950_ = 6.9%.

The small airways (< 2 mm diameter) are the predominant site of airflow limitation in chronic obstructive pulmonary disease (COPD) [1]. Emphysema and large airway disease, two other major pathologies in COPD, can be visualized using chest CT scans. However, the small airways cannot be directly imaged using current CT scan technology [2]. Traditionally, gas trapping on pulmonary function testing, based on the ratio of either residual volume (RV) or inspiratory capacity to total lung capacity (TLC), has been used as a surrogate for small airway disease. Given the marked heterogeneity among COPD subjects and the increasingly widespread use of chest CT scans in smokers with and without COPD [3], there is a need to identify an accurate marker of small airway disease on chest CT scans, which may complement measures of small airway disease derived from traditional lung function testing. The percent of lung voxels with attenuation below a specific threshold, such as −856 Hounsfield units (HU), on expiratory chest CT scans has been proposed as an indicator for gas trapping in subjects with asthma [4]. In subjects with COPD, this threshold method fails to distinguish gas trapping due to emphysema from gas trapping due to small airway disease (Figure 1).

Several alternative gas trapping measures have been proposed in subjects with COPD based on quantitative CT analysis [5-10]. These prior studies have generally had limited sample sizes, with few subjects with either normal lung function or severe COPD. Differentiation of gas trapping due to small airway disease from emphysematous gas trapping is especially important in subjects with more severe COPD, who generally have more emphysema. A recently published lung cancer screening study from the Netherlands included inspiratory and expiratory chest CT scans on a large sample of control smokers and subjects with COPD, showing the independent ability of CT emphysema and CT gas trapping measures to predict COPD status defined by spirometry [11]. However, COPD subjects were generally mild to moderate in severity, with only a limited representation of subjects with severe COPD.

The Genetic Epidemiology of COPD Study (COPDGene) is a large observational study of over 10,000 current and former smokers, including subjects without airflow obstruction and subjects across the full range of COPD severity [12]. We hypothesized that quantitative analysis of paired inspiratory and expiratory chest CT scans in COPDGene can be used to define indicators of small airway disease that are superior to expiratory CT imaging alone as predictors of lung function and other COPD-related traits in smokers across a full range of lung function values from normal to very severe COPD.

## Methods

### Study subjects

COPDGene recruited subjects at 21 clinical centers across the U.S [12]. Subjects were non-Hispanic whites or non-Hispanic African Americans ages 45–80, with a smoking history of at least 10 pack-years. Subjects with other diagnosed lung diseases except for asthma were excluded. Subjects underwent a standard study visit which included spirometry before and after albuterol administration, measurement of exercise capacity using a six-minute walk test, questionnaires to assess medical history, respiratory symptoms and medication use, and a blood sample. The modified Medical Research Council (MMRC) scale was used to quantify dyspnea [13]. Disease-related quality of life was assessed by the St. George’s Respiratory Questionnaire (SGRQ) [14]. In COPDGene, COPD was defined and staged according to GOLD criteria [15], despite potential limitations of a fixed FEV_1_/FVC ratio < 0.7 [16]. Study protocols and questionnaires are available at http://www.copdgene.org. The COPDGene data release version 16-April-2012 was used for analysis. Subjects provided written informed consent. The study was performed in accordance with the Declaration of Helsinki and was approved by the Institutional Review Boards at Partners Healthcare and all participating centers.

### Chest CT scans

Subjects underwent volumetric CT scans of the chest at full inspiration (standard dose = 200mAs) and at end-tidal expiration (low dose = 50 mAs). Detailed CT protocols have been previously published [12] and are available online at http://www.copdgene.org. CT scans were subjected to a standard quality control procedure. Subjects with missing or failed inspiratory or expiratory chest CT scans were from this analysis. Computerized image analysis was performed with 3D SLICER software (http://www.slicer.org) [17,18]. Emphysema was quantified by the percent of the lung voxels on inspiratory CT scan with attenuation < −950 HU (Insp_−950_) [19]. The square root of wall area of a hypothetical airway with 10 mm internal perimeter (SRWA Pi10) [20], measured using VIDA software (http://www.vidadiagnostics.com), was available in a minority of subjects. Small airway measures on chest CT scan were defined as follows:

1. *Expiratory*_*−856*_*(Exp*_*−856*_*)* is defined as the percent of the lung voxels with attenuation < −856HU on the expiratory CT images [4]. This has been called “percent gas trapping” in previous COPDGene publications [21-23].

2. *Expiratory to Inspiratory Ratio of Mean Lung Attenuation (E/I MLA)* is defined as the ratio of mean lung attenuation from the density histograms on expiratory and inspiratory scans [5-7,9,10].

3. *Relative Volume Change*_*-856 to −950*_*(RVC*_*856-950*_*)* is defined as the difference between the expiratory and inspiratory values for relative lung volumes, which is the limited lung volume with attenuation between −856 to 950HU divided by the lung volume without emphysema, according to Matsuoka et al. [8] Relative lung volume on expiratory or inspiratory scan is expressed as:

lung volume with attenuation between −856 and -950HU/

lung volume with attenuation > −950HU

4. *Residuals* are the residuals from the linear regression of Expiratory_−856_ on Inspiratory_−950_. This statistical approach was used to regress out the effect on emphysema on the Expiratory_−856_ measure of gas trapping.

Emphysema was considered absent in subjects with values for Insp_−950_ < 5% in ex-smokers and < 4% in current smokers, to account for the fact that the increased lung density in current smokers results in a decrease in emphysema index [24]. Severe emphysema was defined by Insp_−950_ > 15% in ex-smokers and > 14% in current smokers. Total Lung Capacity (TLC) and Functional Residual Capacity (FRC) were determined from measurement of lung volumes on chest CT scans at full inspiration and relaxed exhalation, respectively [25].

### Statistical analysis

Pearson correlations were used to describe correlations among the small airway measures and between small airway measures and clinical variables. Linear regression models were used to describe the relative contributions of emphysema and small airway disease to lung function and selected clinical parameters. Linear regression models were adjusted for clinically relevant covariates determined *a priori*. Independent variables, including CT emphysema and small airway parameters, were standardized to mean = 0 and standard deviation = 1 in the regression models. The percent of variation explained (R^2^) was used to assess the explanatory ability of a regression model. P-value < 0.05 represents statistical significance. Statistical analyses were conducted using SAS version 9.2 (SAS Institute, Cary, NC).

### Reproducibility data

Thirty-two subjects were inadvertently enrolled twice into COPDGene. Eighteen of those subjects had inspiratory and expiratory chest CT scans performed with the same protocol and passing quality control which were analyzed with 3D SLICER. The eighteen duplicate scans were separated by a median of 224 days (range 38–550). Spearman correlations were used to compare the gas trapping measures in these subjects at the two time points.

### Role of the funding source

The study sponsors had no role in study design; in the collection, analysis, and interpretation of data; in the writing of the report; and in the decision to submit the paper for publication.

## Results

COPDGene enrolled 10,300 subjects, including 108 non-smoker controls who were excluded from this analysis. Table 1 shows the characteristics of the 8517 current and former smokers with complete inspiratory and expiratory chest CT data. Study subjects encompassed the range of COPD severity across all GOLD stages [15]. Approximately 12% of subjects were unclassified by GOLD, with reduced FEV_1_, but normal FEV_1_/FVC ratio; the GOLD-unclassified subjects in COPDGene have been described previously [26]. Surprisingly, 4% of subjects with severe emphysema on chest CT scan had only mild airflow obstruction on spirometry (GOLD1). Conversely, over 4% of subjects without emphysema had severe or very severe airflow obstruction (GOLD 3–4).

**Table 1:** COPDGene study subjects

	**All subjects**	**Severe emphysema‡**	**No emphysema§**
N	8517	1188	5769
Age	59.6 (9.0)	65.5 (7.7)	57.6 (8.6)
Sex			
Male	4535 (53.2%)	711 (59.8%)	2849 (49.4%)
Female	3982 (46.8%)	477 (40.2%)	2920 (50.6%)
Race			
African American	2668 (31.3%)	190 (16.0%)	2156 (37.4%)
White	5849 (68.7%)	998 (84.0%)	3613 (62.6%)
Current smoking	4463 (52.4%)	259 (21.8%)	3547 (61.5%)
Pack-years of smoking	44.3 (24.8)	55.4 (27.8)	40.8 (22.6)
GOLD Stage	[54 missing]†	[3 missing]†	[40 missing]†
0	3674 (43.4%)	17 (1.4%)	3241 (56.6%)
1	668 (7.9%)	47 (4.0%)	414 (7.2%)
2	1636 (19.3%)	263 (22.2%)	870 (15.2%)
3	965 (11.4%)	458 (38.7%)	217 (3.8%)
4	502 (5.9%)	396 (33.4%)	33 (0.6%)
Unclassified*	1018 (12.0%)	4 (0.3%)	954 (16.7%)

Non-smokers were excluded. Mean (SD) or N (%) is shown.

*Unclassified: FEV_1_ < 80% predicted with FEV_1_/FVC > 0.7.

†GOLD Stage is missing in subjects whose spirometry failed quality control.

‡Severe emphysema defined by Insp_-950HU_ > 15% ex-smokers, > 14% current smokers.

§No emphysema defined by Insp_-950HU_ < 5% ex-smokers, < 4% current smokers.

**Figure 2:**
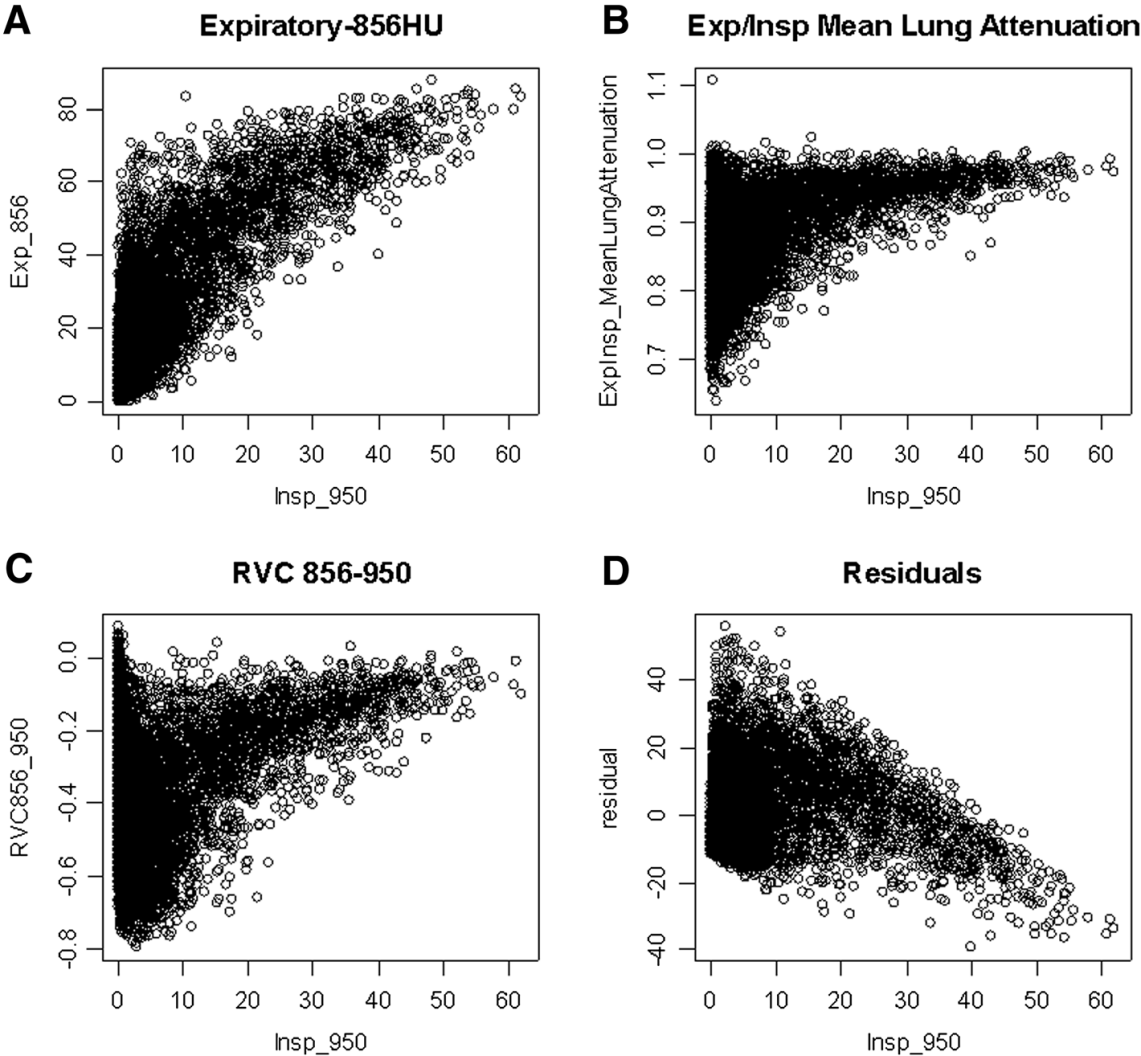
**Scatterplots of four gas trapping measurements vs. percent emphysema (see Methods for definitions).**

Among the quantitative CT measures, Exp_−856_ was most highly correlated with percent emphysema (Table 2, Figure 2). E/I MLA and RVC_856-950_ were significantly but less strongly correlated, while the residuals are completely uncorrelated with Insp_−950_, by definition. The small airway parameters showed varying degrees of correlations among themselves. Correlations between the small airway measures and “medium-sized” airway disease indicated by SRWA Pi10 are weak, except for the correlation with RVC_856-950_. In 1188 subjects with severe emphysema, E/I MLA was less strongly correlated with percent emphysema than were Exp_−856_ and RVC_856-950_ (r = 0.40 for E/I MLA, r = 0.62 for Exp_−856_, r = 0.51 for RVC_856-950_; all p < 0.0001). E/I MLA was only weakly correlated with Insp_−950_ in subjects without emphysema, while Exp_−856_ and RVC_856-950_ were more highly correlated in this group (r = 0.12 for E/I MLA, r = 0.50 for Exp_−856_, r = −0.33 for RVC_856-950_; all p < 0.0001).

**Table 2:** Correlations between quantitative CT measures in COPDGene subjects

			**Correlations**					
	**N**	**Mean(SD)**	**%emph Insp **_**-950**_	**Exp **_**-856**_	**E/I mean lung atten.**	**RVC **_**856–950**_	**Residuals**	**SWRA Pi10**
%emphysema Insp _-950_	8517	6.2 (9.7)	--					
%Exp _-856_	8517	21.9 (19.9)	0.83†	--				
E/I mean lung attenuation	8517	0.87 (0.07)	0.51†	0.80†	--			
RVC _856–950_	8517	−0.37 (0.18)	0.37†	0.48†	0.58†	--		
Residuals	8517	0 (11.2)	0	0.56†	0.67†	0.32†	--	
SRWA Pi10 (mm)	2867	3.75 (0.12)	0.06*	0.13†	0.23†	0.46†	0.15†	--

Pearson correlation coefficients are shown.

*p < 0.01.

†p < 0.0001.

Correlations between the small airway measures, lung function, and other COPD-related traits are shown in Tables 3 and 4. In all subjects, Exp_−856_ showed the highest correlations with lung function measurements, including FEV_1_, FEV_1_/FVC and FEF_25-75_, a putative small airway disease marker from spirometry. E/I MLA showed a similar correlation as Exp_−856_ with FEF_25-75_ in all subjects. FRC/TLC ratio, a measure of hyperinflation based on chest CT scan-derived lung volumes was most highly correlated with E/I MLA. Exp_−856_, E/I MLA and RVC_856-950_ showed similar correlations with SGRQ and MMRC dyspnea score; RVC_856-950_ was most strongly correlated with 6-minute walk distance (6MWD). Residuals showed the weakest correlations with all tested traits.

**Table 3:** Correlations between gas trapping measures and quantitative outcomes in all subjects

			**Correlations**			
	**N**	**Mean (SD)**	**Exp**_**−856**_	**E/I MLA**	**RVC**_**856-950**_	**Residual**
FEV_1_ % predicted	8463	76.6 (25.5)	−0.69*	−0.60*	−0.54*	−0.33*
FVC % predicted	8463	87.2 (18.2)	−0.33*	−0.32*	−0.44*	−0.18*
FEV_1_/FVC	8463	0.67 (0.16)	−0.82*	−0.67*	−0.41*	−0.37*
FEF_25-75_	8463	1.75 (1.25)	−0.60*	−0.59*	−0.38*	−0.36*
FRC/TLC ratio	8517	0.58 (0.13)	0.65*	0.89*	0.76*	0.65*
6MWD	8407	1363 (397)	−0.32*	−0.34*	−0.46*	−0.18*
Exacerbation frequency	8517	0.4 (0.9)	0.26*	0.22*	0.19*	0.12*
SGRQ total	8517	27.0 (22.8)	0.39*	0.36*	0.43*	0.20*
MMRC dyspnea	8506	1.3 (1.4)	0.36*	0.32*	0.42*	0.15*

Pearson correlation coefficients are shown.

*p < 0.0001.

**Table 4:** **Correlations between gas trapping measures and quantitative outcomes in subjects with severe emphysema (Insp**_**-950HU**_ **> 15% ex-smokers, Insp**_**-950HU**_ **> 14% current smokers)**

			**Correlations**			
	**N**	**Mean (SD)**	**Exp**_**−856**_	**E/I MLA**	**RVC**_**856-950**_	**Residual**
FEV_1_ % predicted	1185	41.2 (19.9)	−0.69‡	−0.73‡	−0.73‡	−0.16‡
FVC % predicted	1185	75.7 (21.1)	−0.49‡	−0.56‡	−0.57‡	−0.17‡
FEV_1_/FVC	1185	0.40 (0.12)	−0.66‡	−0.62‡	−0.62‡	−0.08*
FEF_25-75_	1185	0.42 (0.38)	−0.56‡	−0.61‡	−0.61‡	−0.18‡
FRC/TLC ratio	1188	0.69 (0.12)	0.80*	0.93*	0.87*	0.44*
6MWD	1135	1116 (404)	−0.31‡	−0.40‡	−0.43‡	−0.01^NS^
Exacerbation frequency	1188	0.8 (1.2)	0.10†	0.12‡	0.13‡	−0.02^NS^
SGRQ total	1188	44.9 (19.9)	0.32‡	0.41‡	0.43‡	0.09*
MMRC dyspnea	1183	2.5 (1.3)	0.34‡	0.38‡	0.42‡	0.01^NS^

Pearson correlation coefficients are shown.

*p < 0.01.

†p < 0.001.

‡p < 0.0001.

NS = not significant (p > 0.05).

In subjects with severe emphysema (Table 4), E/I MLA and RVC_856-950_ were most highly correlated with all tested traits, except for FEV_1_/FVC which showed a slightly higher correlation with Exp_−856_. Again, residuals showed the poorest correlations with lung function, 6-minute walk distance and symptoms. In subjects without significant emphysema (Additional file 1: Table S1), RVC_856-950_ or E/I MLA generally showed the strongest correlations. E/I MLA was most highly correlated with FEV_1_/FVC, FEF_25-75_ and FRC/TLC ratio. Residuals generally showed the weakest correlations with all tested outcomes.

Table 5 shows the joint effects of emphysema (Insp_−950_) and each of three small airway measures (Exp_−856_, E/I MLA, and RVC_856-950_) on the COPD-related traits FEV_1_, FEV_1_/FVC, FEF_25-75_, 6-minute walk distance and SGRQ. Residuals were not used in the regression analysis due to consistently weak correlations in the earlier analyses. Emphysema and gas trapping variables were standardized, so the regression coefficients reflect the effect of a one standard deviation change in each CT variable. Based on percent variation explained (R^2^), all three small airway measures were equally predictive of FEV_1_. The effects of emphysema and gas trapping on FEV_1_ were equivalent when either E/I MLA or RVC_856-950_ were used as the gas trapping measure, whereas the gas trapping variable exerted more than three times the effect of emphysema (−0.45 L vs. -0.11 L) in the model using Exp_−856_. Models including either Exp_−856_ or E/I MLA explained more of the variation in FEV_1_/FVC and in FEF_25-75_ than the model including RVC_856-950_. The three gas trapping variables were similar in models predicting 6MWD and SGRQ score in all subjects.

**Table 5:** Regression models for lung function, exercise capacity and symptoms in all subjects

A. Linear regression for FEV_1_ (L)						
	Exp_−856_		E/I MLA		RVC_856-950_	
*Independent variables*	β*	p-value	β*	p-value	β*	p-value
% emphysema (Insp_−950_)	−0.11	< 0.0001	−0.31	< 0.0001	−0.29	< 0.0001
Gas Trapping variable†	−0.45	< 0.0001	−0.30	< 0.0001	−0.30	< 0.0001
*Model fit statistics*						
R^2^	0.67		0.68		0.68	
B. Linear regression for FEV_1_/FVC						
	Exp_−856_		E/I MLA		RVC_856-950_	
*Independent variables*	β*	p-value	β*	p-value	β*	p-value
% emphysema (Insp_−950_)	−0.03	< 0.0001	−0.09	< 0.0001	−0.10	< 0.0001
Gas Trapping variable†	−0.10	< 0.0001	−0.06	< 0.0001	−0.03	< 0.0001
*Model fit statistics*						
R^2^	0.73		0.70		0.64	
C. Linear regression for FEF_25-75_						
	Exp_−856_		E/I MLA		RVC_856-950_	
*Independent variables*	β*	p-value	β*	p-value	β*	p-value
% emphysema (Insp_−950_)	0.0002	0.99	−0.29	< 0.0001	−0.38	< 0.0001
Gas Trapping variable†	−0.68	< 0.0001	−0.51	< 0.0001	−0.24	< 0.0001
*Model fit statistics*						
R_2_	0.50		0.52		0.45	
D. Linear regression for 6-minute walk distance (ft).						
	Exp_−856_		E/I MLA		RVC_856-950_	
*Independent variables*	β*	p-value	β*	p-value	β*	p-value
% emphysema (Insp_−950_)	−47.8	< 0.0001	−46.3	< 0.0001	−37.3	< 0.0001
Gas Trapping variable†	−3.7	0.6	−22.0	< 0.0001	−51.8	< 0.0001
*Model fit statistics*						
R^2^	0.44		0.44		0.45	
E. Linear regression for SGRQ Total score.						
	Exp_−856_		E/I MLA		RVC_856-950_	
*Independent variables*	β*	p-value	β*	p-value	β*	p-value
% emphysema (Insp_−950_)	1.3	0.0004	2.8	< 0.0001	2.7	< 0.0001
Gas Trapping variable†	3.2	< 0.0001	1.9	< 0.0001	1.7	< 0.0001
*Model fit statistics*						
R^2^	0.42		0.42		0.42	

All models are adjusted for age, sex, race, clinical center, current smoking status, pack-years of smoking, height, weight, Siemens 64 scanner. Models for 6-minute walk distance (D) and SGRQ score (E) were additionally adjusted for FEV1% predicted.

*Independent variables standardized to mean = 0 and SD = 1. β = change in dependent variable, e.g. FEV_1_ (L), for each SD change in independent variable.

†Either Exp_−856_, E/I mean lung attenuation ratio, or RVC_856-950_.

In subjects with severe emphysema (Table 6), models with E/I MLA or RVC_856-950_ were better predictors of FEV_1_ than was Exp_−856_. Only the model using E/I MLA captured significant effects from both emphysema and gas trapping variables. All three small airway measures yielded similarly predictive models for FEV_1_/FVC, FEF_25-75_, 6MWD, and SGRQ. In the analysis of 6MWD, use of Exp_−856_ led to a significant positive effect of gas trapping, meaning an increase in the gas trapping variable corresponded to an increase in 6MWD among severe emphysema subjects. The 6MWD models with E/I MLA or RVC_856-950_ captured significant effects of emphysema only, with an expected direction of effect, namely reduction in 6MWD. Both E/I MLA and RVC_856-950_ identified statistically significant effects of gas trapping on SGRQ score in subjects with severe emphysema.

**Table 6:** Regression models for lung function, exercise capacity and symptoms in subjects with severe emphysema

A. Linear regression for FEV_1_ (L)						
	Exp_−856_		E/I MLA		RVC_856-950_	
*Independent variables*	β*	p-value	β*	p-value	β*	p-value
% emphysema (Insp_−950_)	0.01	0.5	−0.08	< 0.0001	−0.01	0.3
Gas Trapping variable†	−0.46	< 0.0001	−0.43	< 0.0001	−0.46	< 0.0001
*Model fit statistics*						
R^2^	0.63		0.68		0.67	
B. Linear regression for FEV_1_/FVC						
	Exp_−856_		E/I MLA		RVC_856-950_	
*Independent variables*	β*	p-value	β*	p-value	β*	p-value
% emphysema (Insp_−950_)	−0.01	0.004	−0.03	< 0.0001	−0.02	< 0.0001
Gas Trapping variable†	−0.08	< 0.0001	−0.07	< 0.0001	−0.07	< 0.0001
*Model fit statistics*						
R^2^	0.52		0.52		0.49	
C. Linear regression for FEF_25-75_						
	Exp_−856_		E/I MLA		RVC_856-950_	
*Independent variables*	β*	p-value	β*	p-value	β*	p-value
% emphysema (Insp_−950_)	0.01	0.3	−0.04	0.0001	−0.004	0.7
Gas Trapping variable†	−0.24	< 0.0001	−0.23	< 0.0001	−0.24	< 0.0001
*Model fit statistics*						
R^2^	0.46		0.50		0.49	
D. Linear regression for 6-minute walk distance (ft).						
	Exp_−856_		E/I MLA		RVC_856-950_	
*Independent variables*	β*	p-value	β*	p-value	β*	p-value
% emphysema (Insp_−950_)	−66.6	< 0.0001	−51.7	< 0.0001	−46.6	0.0002
Gas Trapping variable†	41.4	0.01	−3.4	0.8	−22.4	0.2
*Model fit statistics*						
R^2^	0.41		0.41		0.41	
E. Linear regression for SGRQ Total score.						
	Exp_−856_		E/I MLA		RVC_856-950_	
*Independent variables*	β*	p-value	β*	p-value	β*	p-value
% emphysema (Insp_−950_)	0.9	0.2	0.9	0.1	0.1	0.8
Gas Trapping variable†	1.1	0.2	3.0	0.0001	4.4	< 0.0001
*Model fit statistics*						
R^2^	0.34		0.35		0.36	

All models are adjusted for age, sex, race, clinical center, current smoking status, pack-years of smoking, height, weight, Siemens 64 scanner. Models for 6-minute walk distance (D) and SGRQ score (E) were additionally adjusted for FEV1% predicted.

*Independent variables standardized to mean = 0 and SD = 1. β = change in dependent variable, e.g. FEV_1_ (L), for each SD change in independent variable.

†Either Exp_−856_, E/I mean lung attenuation ratio, or RVC_856-950_.

In subjects without significant emphysema, models including each of the small airway measures explained similar fractions of the variation in the COPD-related traits, with the exception of RVC_856-950_ predicting FEV_1_/FVC (Additional file 2: Table S2). In these subjects, quantitative emphysema measurements were not significantly associated with FEV_1_, FEV_1_/FVC or 6MWD when E/I MLA was used as the gas trapping indicator.

In the eighteen subjects with repeat quantitative CT data, the gas trapping measures from the two time points were significantly correlated within each subject: Exp_−856_ ρ = 0.79, p < 0.0001; E/I MLA ρ = 0.67, p = 0.002; RVC_856-950_ ρ = 0.54, p = 0.02.

## Discussion

In the largest cohort of smokers with quantitative chest CT data published to date, we evaluated four variables to measure gas trapping as a surrogate for small airway disease, since the airways of interest are below the resolution of CT imaging. Three of the variables were defined in an effort to separate gas trapping due to small airway disease from gas trapping due to emphysema. The residuals from regression of expiratory air trapping on emphysema showed consistently poor correlations and were not tested in multivariable models. In general, the other three measures – percent of lung with attenuation < −856HU on expiratory chest CT scan, the expiratory to inspiratory ratio of mean lung attenuation, and the relative volume change_856-950_ – were similarly predictive of lung function, exercise capacity and quality of life when all subjects were considered together (Table 5). However, in subjects with severe emphysema, the two measures that utilized paired inspiratory and expiratory CT scans – E/I MLA and RVC_856-950_ – yielded more predictive associations (Table 6). The paired measures performed adequately in subjects without significant emphysema as well (Additional file 2: Table S2). The variables had reasonable reproducibility in a small subset of subjects with duplicate chest CT scans, although repeat correlations were stronger Exp_−856_ than for E/I MLA or RVC_856-950_, since the latter two measures incorporate variability from both inspiratory and expiratory acquisitions.

Assessment of low attenuation areas on expiratory chest CT scans was initially described as a marker for gas trapping in studies of asthma [27]. The Severe Asthma Research Program proposed the threshold of -856HU [4]. As opposed to asthma, in COPD both emphysema and gas trapping from small airway disease may lead to increased values for Exp_−856_.

Several groups have examined paired inspiratory and expiratory CT scan measures of gas trapping in COPD. The majority of papers have used the expiratory to inspiratory ratio of mean lung attenuation, which has also been referred to as the ratio of mean lung density [5-7,10]. Matsuoka and colleagues defined RVC_850-950_[8], similar to RVC_856-950_ in the current analysis. These previous studies have found good correlations between pulmonary function measures and gas trapping. However, the sample sizes in the published papers have generally been small and most of the papers have only considered a single gas trapping metric. Mets and colleagues compared several different gas trapping measures in 248 subjects [9]. Similar to our analysis, they found that the two paired measures – E/I MLA and RVC_860-950_ in their study – had the strongest correlations with lung function. We confirmed these findings in a much larger cohort, and found parallel correlations with important clinical outcomes including exercise capacity and quality of life. Most of the previous papers performed expiratory CT scans at full expiration (RV), whereas COPDGene scans were performed at end-tidal expiration, corresponding to FRC.

A recently published Dutch lung cancer screening study acquired low-dose inspiratory and end-expiratory chest CT scans in 1140 male smokers at a single center [11]. A multivariable model which included both CT emphysema (Insp_−950_) and E/I MLA as a marker of gas trapping was highly accurate in predicting COPD. Although 437 (38%) subjects had COPD based on spirometry, only 25 subjects had severe COPD (GOLD 3) and none had very severe COPD (GOLD 4).

The large number of subjects with quantitative analysis of inspiratory and expiratory chest CT scans is one of the strengths of the COPDGene study. The sample size in COPDGene is nearly ten times as large as the Dutch cancer screening study [11]. COPDGene included smokers with normal lung function and with the full range of lung function impairment, in contrast to previous papers. Including subjects with severe and very severe COPD (GOLD 3–4) allowed for the analysis in subjects with severe emphysema, though emphysema severity did not completely correlate with GOLD stage. The extensive clinical characterization in COPDGene allowed us to select a range of COPD-related traits including spirometry, exercise capacity and disease related quality of life. Subjects in COPDGene were enrolled at multiple clinical centers and underwent CT scans on different scanner models, yet we were able to find robust associations which were applicable across the multi-center study.

Ideally, one would test the CT measures of small airway disease against physiologic measures that have been traditionally used to measure small airway disease, including the RV/TLC ratio. However, lung volumes were not measured by pulmonary function testing in COPDGene, but were assessed on chest CT scans. E/I MLA showed the strongest correlation with the CT-derived FRC/TLC ratio, a marker of hyperinflation. Study subjects were given standard instructions for breath-holds at full inspiration (TLC) and end-tidal expiration (FRC), but the chest CT scans did not use spirometric gating to ensure compliance. The CT scanning protocol in COPDGene more accurately reflects clinical practice and still provides important information in the research setting. We did find significant correlations with FEF_25-75_, a putative small airway disease measurement on spirometry. Measurement of carbon monoxide diffusing capacity (DLCO) can be used as an indicator of emphysema on pulmonary function testing; however DLCO was not measured in the COPDGene Study. The CT measures may complement standard lung function testing in the evaluation of COPD patients.

Besides histology, direct visualization of the small airways would be the optimal metric for small airway disease in smokers. This can be performed *ex vivo* using microCT [28], but is not feasible in living patients. There is substantial literature using measurements of airway disease in larger airways, such as SRWA Pi10 [20]. Papers from COPDGene have found correlations between segmental and subsegmental airway wall measures and clinical traits including exacerbations [29], chronic bronchitis [30], pulmonary function [31] and quality of life [32]. These approaches rely on the assumption that similar pathological processes are occurring in the small airways and the more proximal airways. At present, the choice of the best airway wall measurement is not clear, and any such measure on the larger airways is primarily a surrogate for small airway disease. Pairing standard inspiratory chest CT scans with low dose expiratory scans can provide an alternative marker for airflow obstruction in the small airways in smokers, yet most large COPD studies do not acquire expiratory CT scans [33,34].

## Conclusions

We have shown that two previously-described gas trapping measures based on paired inspiratory and expiratory chest CT scans (E/I MLA and RVC_856-950_) may serve as markers of small airway disease in smokers, including subjects with severe and very severe COPD. Although we cannot claim that one measure is superior to the other, the expiratory to inspiratory ratio of mean lung attenuation may be more straightforward than RVC_856-950_, and has more support in the published COPD literature. For clinical applicability, it will be important to define thresholds for these gas trapping parameters that delineate normal from small airway disease; a large sample of never smokers with normal lung function is necessary for this effort. Additionally, thresholds to define airway-predominant vs. emphysema-predominant COPD subtypes are also desirable. The current data do not allow for assessment of changes in these measures with treatment, an important area for future investigation. Large studies of smokers will have to include expiratory CT scans to further these efforts. In COPDGene and future studies, these gas trapping variables can be used in genetic analyses, to better understand the pathobiology of COPD subtypes.

## Competing interests

Dr. Hersh has received lecture fees from Novartis. Dr. Han has served as a consultant for Boehringer Ingelheim, Pfizer, GSK, Medimmune, Novartis, Grifols Therapeutics, and United Biosource Corporation. She has received royalties from UpToDate. She has developed educational presentations for National Association for Continuing Education and WebMD. Dr. Lynch has received grant support from Siemens and Centocor. He has served as a consultant for Perceptive Imaging, Intermune and Gilead.

Dr. Make has served on advisory boards for Forest, AstraZeneca, Novartis, Coviden, Breathe, Merck, Sunovion, Boehringer Ingelheim, MedImmune, Ikaria, and Novartis. He has served as a consultant for Astellas. He reports grant support from AstraZeneca, GlaxoSmithKline, NABI, Boehringer Ingelheim, Sunovion, and Forest. He has received lecture fees from GlaxoSmithKline, Boehringer Ingelheim, Pfizer, and Forest. He has received royalties from UpToDate. Dr. Newell has served as a consultant for VIDA Diagnostics. Dr. Sciurba has participated in consulting for GSK, AstraZeneca and Pfizer and has received research grant funding from the NIH, GSK, BI, Pfizer, Forest and Actelion. Dr. Silverman has served as a consultant for GlaxoSmithKline, AstraZeneca, and Merck. He has received grant support from GlaxoSmithKline. He has received lecture fees from GlaxoSmithKline and AstraZeneca.

Drs. Washko, San José Estépar, Lutz, Friedman, Hokanson, Judy, Marchetti and Crapo do not report any competing interests.

## Authors’ contributions

Study concept and design: CPH, GRW, PJF, MKH, JEH, PFJ, DAL, BJM, NM, JDN, FCS, JDC, EKS. Data collection: CPH, GRW, RSJE, PJF, MKH, JEH, PFJ, DAL, BJM, NM, JDN, FCS, JDC, EKS. Data analysis: CPH, RSJE, SL. Manuscript drafting: CPH. Manuscript revision and final approval: All authors.

## Supplementary Material

Additional file 1: Table S1 Correlations between gas trapping measures and quantitative outcomes in subjects without emphysema (Insp_-950_ < 5% ex-smokers, < 4% current smokers). Click here for file

Additional file 2: Table S2 Regression models for lung function, exercise capacity and symptoms in subjects without emphysema. Click here for file
