# Supplementary material for: Paired inspiratory-expiratory chest CT scans to assess for small airways disease in COPD
Source: Respir Res. 2013 Apr 8;14(1):42. doi: 10.1186/1465-9921-14-42 (PMC3627637; doi:10.1186/1465-9921-14-42)
Supplement: Additional file 1: Table S1 — Correlations between gas trapping measures and quantitative outcomes in subjects without emphysema (Insp-950 < 5% ex-smokers, < 4% current smokers). [file 1465-9921-14-42-S1.doc]

Additional file 1: Table S1: Correlations between gas trapping measures and quantitative outcomes in subjects without emphysema (Insp_-950_ <5% ex-smokers, <4% current smokers)

Pearson correlation coefficients are shown.

|  |  |  | Correlations | | | |
| --- | --- | --- | --- | --- | --- | --- |
|  | N | Mean (SD) | Exp_-856_ | E/I MLA | RVC_856-950_ | Residual |
| FEV_1_ % predicted | 5729 | 85.2 (19.1) | -0.27† | -0.31† | -0.32† | -0.28† |
| FVC % predicted | 5729 | 89.2 (16.2) | -0.07† | -0.13† | -0.33† | -0.09† |
| FEV_1_/FVC | 5729 | 0.74 (0.10) | -0.48† | -0.43† | -0.05* | -0.45† |
| FEF_25-75_ | 5729 | 2.14 (1.17) | -0.34† | -0.40† | -0.17† | -0.33† |
| FRC/TLC Ratio | 5769 | 0.56 (0.12) | 0.62† | 0.89† | 0.68† | 0.69† |
| 6MWD | 5724 | 1411 (370) | -0.13† | -0.20† | -0.39† | -0.15† |
| Exacerbation frequency | 5769 | 0.3 (0.7) | 0.10† | 0.11† | 0.09† | 0.10† |
| SGRQ total | 5769 | 22.8 (21.3) | 0.12† | 0.16† | 0.30† | 0.14† |
| MMRC dyspnea | 5768 | 1.1 (1.4) | 0.08† | 0.12† | 0.29† | 0.10† |

*p<0.001

†p<0.0001
