# Supplementary material for: Paired inspiratory-expiratory chest CT scans to assess for small airways disease in COPD
Source: Respir Res. 2013 Apr 8;14(1):42. doi: 10.1186/1465-9921-14-42 (PMC3627637; doi:10.1186/1465-9921-14-42)
Supplement: Additional file 2: Table S2 — Regression models for lung function, exercise capacity and symptoms in subjects without emphysema. [file 1465-9921-14-42-S2.doc]

Additional file 2: Table S2: Regression models for lung function, exercise capacity and symptoms in subjects without emphysema.

All models are adjusted for age, sex, race, clinical center, current smoking status, pack-years of smoking, height, weight, Siemens 64 scanner.

1. Linear Regression for FEV_1_ (L)

|  | Exp_-856_ | | E/I MLA | | RVC_856-950_ | |
| --- | --- | --- | --- | --- | --- | --- |
| *Independent variables* | β* | p-value | β* | p-value | β* | p-value |
| % emphysema (Insp_-950_) | -0.06 | <0.0001 | -0.01 | 0.2 | -0.09 | <0.0001 |
| Gas Trapping variable† | -0.18 | <0.0001 | -0.19 | <0.0001 | -0.23 | <0.0001 |
|  | | | | | | |
| *Model fit statistics* |  | |  | |  | |
| R^2^ | 0.57 | | 0.58 | | 0.59 | |

*Independent variables standardized to mean=0 and SD=1. β=change in FEV_1_ (L) for each SD change in independent variable.

†Either Exp_-856_, E/I mean lung attenuation ratio, or RVC_856-950_.

1. Linear Regression for FEV_1_/FVC

|  | Exp_-856_ | | E/I MLA | | RVC_856-950_ | |
| --- | --- | --- | --- | --- | --- | --- |
| *Independent variables* | β* | p-value | β* | p-value | β* | p-value |
| % emphysema (Insp_-950_) | -0.01 | <0.0001 | -0.03 | <0.0001 | -0.04 | <0.0001 |
| Gas Trapping variable† | -0.04 | <0.0001 | -0.04 | <0.0001 | -0.02 | <0.0001 |
|  | | | | | | |
| *Model fit statistics* |  | |  | |  | |
| R^2^ | 0.33 | | 0.34 | | 0.26 | |

*Independent variables standardized to mean=0 and SD=1. β=change in FEV_1_ (L) for each SD change in independent variable.

†Either Exp_-856_, E/I mean lung attenuation ratio, or RVC_856-950_.

1. Linear regression for FEF_25-75_

|  | Exp_-856_ | | E/I MLA | | RVC_856-950_ | |
| --- | --- | --- | --- | --- | --- | --- |
| *Independent variables* | β* | p-value | β* | p-value | β* | p-value |
| % emphysema (Insp_-950_) | -0.07 | <0.0001 | -0.18 | <0.0001 | -0.30 | <0.0001 |
| Gas Trapping variable† | -0.32 | <0.0001 | -0.38 | <0.0001 | -0.26 | <0.0001 |
|  | | | | | | |
| *Model fit statistics* |  | |  | |  | |
| R^2^ | 0.34 | | 0.37 | | 0.32 | |

*Independent variables standardized to mean=0 and SD=1. β=change in FEV_1_ (L) for each SD change in independent variable.

†Either Exp_-856_, E/I mean lung attenuation ratio, or RVC_856-950_.

1. Linear regression for 6-minute walk distance (ft). Additionally adjusted for FEV_1_ % predicted.

|  | Exp_-856_ | | E/I MLA | | RVC_856-950_ | |
| --- | --- | --- | --- | --- | --- | --- |
| *Independent variables* | β* | p-value | β* | p-value | β* | p-value |
| % emphysema (Insp_-950_) | 11.6 | 0.02 | 6.8 | 0.1 | -11.3 | 0.02 |
| Gas Trapping variable† | -15.2 | 0.004 | -24.4 | <0.0001 | -54.0 | <0.0001 |
|  | | | | | | |
| *Model fit statistics* |  | |  | |  | |
| R^2^ | 0.39 | | 0.40 | | 0.40 | |

*Independent variables standardized to mean=0 and SD=1. β=change in FEV_1_ (L) for each SD change in independent variable.

†Either Exp_-856_, E/I mean lung attenuation ratio, or RVC_856-950_.

1. Linear regression for SGRQ total score. Additionally adjusted for FEV_1_ % predicted.

|  | Exp_-856_ | | E/I MLA | | RVC_856-950_ | |
| --- | --- | --- | --- | --- | --- | --- |
| *Independent variables* | β* | p-value | β* | p-value | β* | p-value |
| % emphysema (Insp_-950_) | 0.6 | 0.07 | 1.2 | <0.0001 | 1.9 | <0.0001 |
| Gas Trapping variable† | 1.5 | <0.0001 | 1.3 | <0.0001 | 1.9 | <0.0001 |
|  | | | | | | |
| *Model fit statistics* |  | |  | |  | |
| R^2^ | 0.31 | | 0.31 | | 0.31 | |

*Independent variables standardized to mean=0 and SD=1. β=change in FEV_1_ (L) for each SD change in independent variable.

†Either Exp_-856_, E/I mean lung attenuation ratio, or RVC_856-950_
